# The effectiveness of public cultural service provision in rural areas of Southwest China: Influencing factors and driving pathways

**DOI:** 10.1371/journal.pone.0342794

**Published:** 2026-02-24

**Authors:** Xi Zhang, Lian Ran

**Affiliations:** 1 School of Marxism, Southwest Jiaotong University, Chengdu, Sichuan, China; 2 School of Social Work and Health Management, Xihua University, Chengdu, Sichuan, China; Zhejiang Shuren University, CHINA

## Abstract

This study explores the influencing factors and driving pathways of rural public cultural service supply effectiveness in Southwest China, addressing the core question, “How does supply effectiveness emerge?” Based on Parsons’ AGIL structural-functional theory, this paper constructs a systematic theoretical framework encompassing “Resource Allocation (A)—Policy Support (I)—Value Shaping (L)—Service Production (G)—Service Effectiveness.” Structural equation modeling was applied to empirically analyze 610 valid questionnaires collected from multiple rural areas in Sichuan Province. Findings reveal that resource allocation serves as the foundational driving force of the system, enhancing service effectiveness primarily through the mediating effects of policy support and service production. Policy support exerts a significant direct effect on value shaping and service effectiveness, but its direct impact on service production is insignificant. Although value shaping does not directly drive service production, it plays a crucial indirect role by enhancing service effectiveness. As the output component of the system, service production directly influences service effectiveness. This study reveals the structured, networked mechanisms that enhance the effectiveness of rural public cultural service supply, providing theoretical and empirical foundations for optimizing supply structures and implementing targeted interventions.

## Introduction

The Rural Revitalization strategy was proposed by China in 2017, with specific implementation guidelines focusing on “prosperous industries, livable ecology, cultural-ethical advancement, effective governance, and improved living standards.” Internationally, concepts and strategies analogous to Rural Revitalization, such as rural development, rural reconstruction, and rural discovery, have been explored in policy and research. Scholarly work in this domain has primarily addressed rural entrepreneurship [1], financial mechanisms [2], community collaboration [3], and policy-driven rural governance [4]. Beyond these socioeconomic dimensions, rural culture is recognized as a key determinant of community health [5] and exerts a multifaceted influence on rural economic and social transformation. Therefore, nurturing rural culture is pivotal in transitioning from traditional to modern rural development paradigms [6]. Theoretically, culture in the context of Rural Revitalization is considered a public good. Its effective provision and management are central to national governance frameworks [7]. The efficient provision of public goods, particularly cultural services, can generate substantial societal benefits and catalyze economic development. This is especially relevant for developing countries, where strategic investment in public services can stimulate economic growth [8]. This study defined the effectiveness of rural public cultural service provision as to the overall degree to which the rural public cultural service delivery system—under specific resource conditions—achieves predetermined systemic objectives. These objectives include meeting farmers’ public cultural needs, ensuring cultural continuity, and fostering social integration. This is accomplished through policy integration and value guidance to generate and deliver public services.

Existing research on rural public cultural service provision primarily focuses on three core dimensions: (1) provision models, with studies exploring the evolutionary path from government-led to multi-stakeholder collaboration [9]; (2) provision challenges, which have revealed widespread difficulties such as uneven resource distribution [10], urban-rural disparities [11], and fiscal and infrastructure shortages [12,13]; and (3) influencing factors, which have identified multiple variables, including collective action [14], audience scale [15], ethnic group differences [16], supply models [17], financial support [18], and talent resources [19]. These studies provide a valuable foundation for understanding the complexity of rural public cultural services.

In summary, existing research provides rich empirical material and partial insights into understanding the supply models, practical challenges, and influencing factors of rural public cultural services. However, a systematic review reveals that research in this field still faces significant limitations in theoretical integration and analytical depth: most studies either focus on single factors such as funding, facilities, or policies, or emphasize descriptive accounts of supply models and challenges. There remains a lack of an integrated theoretical framework capable of simultaneously accommodating multiple core dimensions—including resource foundations, institutional arrangements, value orientations, and service outputs—while systematically elucidating the interactive relationships among them. This results in our understanding of the core question—“How does supply effectiveness emerge?”—often remaining at the level of discrete “factor lists” or linear “causal chains,” failing to reveal the underlying complex, structured systemic dynamics. Therefore, to elucidate the effectiveness of rural public cultural service supply, a more systematic, structural, and interactive theoretical perspective is urgently needed.

The key research gap this study aims to address is how to systematically deconstruct and empirically test the multidimensional synergistic driving mechanism for enhancing the effectiveness of rural public cultural service provision. Specifically, we ask how the four key dimensions—resource allocation, policy support, value shaping, and service production—interconnect, interact, and ultimately jointly drive improvements in service effectiveness. To address this question, the study adopts Parsons’ AGIL structural-functional model as an integrative theoretical framework, constructing a structured theoretical model encompassing “Resource Allocation (A)—Policy Support (I)—Value Shaping (L)—Service Production (G)—Service Effectiveness.” Subsequently, employing structural equation modeling (SEM) as a multivariate statistical method and utilizing survey data from rural areas in Southwest China, we simultaneously examine: (1) the path relationships (direct effects) among the dimensions, and (2) the mediating effects of policy support, value shaping, and service production (indirect effects). This approach transcends linear attribution to reveal the complex, structured network of drivers behind effectiveness improvement, thereby providing empirical evidence and decision-making references for optimizing supply structures and achieving targeted interventions.

### Theoretical framework

The effectiveness of rural public cultural services reflects the outcomes and impacts generated during their provision and serves as a direct indicator of the level of these services. Therefore, examining the factors that enhance service provision efficiency is essential for optimizing the content and improving the quality of rural public cultural services. This study employs the AGIL structural-functional framework to analyze factors that enhance the effectiveness of rural public cultural service provision. The introduction of the AGIL framework serves to bridge the fragmented state of existing research on factors influencing the effectiveness of rural public cultural services, providing a systematic “organizational framework” for such studies. Parsons’ AGIL structural-functional framework posits that for a social system to maintain stability and achieve self-regulation, it must fulfill four functional prerequisites: (1) adaptation, which involves acquiring sufficient resources from the external environment and distributing them within the system to adapt to environmental changes and ensure survival; (2) goal-attainment, which refers to maximizing the use of resources to achieve the system’s objectives; (3) integration, which involves overcoming deviant behavior and maintaining the coordinated functioning of the system’s components; and (4) latent pattern maintenance, which refers to the transmission of societal values to sustain the social value system, ensuring that the underlying patterns of social action are not disrupted [20].

To systematically deconstruct the complex mechanisms underlying the effectiveness of rural public cultural service provision, this study adopts Parsons’ AGIL model as its core analytical framework. Its applicability is grounded in the following four targeted advantages: First, AGIL’s holistic systems perspective aligns with the multidimensional complexity of supply systems. This model requires simultaneous attention to the interactions among four functional subsystems: resource allocation (A), policy support (I), value shaping (L), and service production (G). It comprehensively captures the nested relationships between “policy-resources-value-outputs,” avoiding the limitations of theories like New Public Management, which overemphasizes efficiency, or social capital theory, which tends to focus on single dimensions. Second, its “structure-function” paradigm provides a profound insight into the operational logic of China’s organized supply system. By deconstructing the system into subsystems performing distinct functions, AGIL guides analysis beyond superficial factor enumeration. It examines how resources adapt, administration integrates, culture models, and services achieve objectives, thereby clarifying the deep mechanisms of efficiency generation at the structural level. Moreover, AGIL provides a dedicated theoretical framework for the core intangible dimensions of public cultural services: values and meaning. The “latent pattern maintenance (L)” subsystem within the model is specifically designed to analyze value transmission and cultural identity, enabling the incorporation of “value shaping” as a foundational dimension alongside resources, policies, and outputs. This addresses the shortcomings of theories such as public choice and performance management in this critical dimension. Finally, AGIL’s dynamic equilibrium perspective directly resonates with the practical agenda of “supply-side structural reform.” This model emphasizes that subsystems must maintain dynamic equilibrium and energy exchange, providing a systematic analytical blueprint for diagnosing structural shortcomings in current supply—such as the disconnect between policy integration and service delivery—and for exploring ways to enhance overall effectiveness through resource reallocation, policy optimization, and value restructuring. Therefore, the AGIL model, with its systemic integrity, structural interconnectivity, value inclusivity, and dynamic equilibrium, provides a logically coherent, multidimensional, and highly explanatory theoretical framework for analyzing rural public cultural service systems that encompass administrative, cultural, and social dimensions.

**Fig 1:**
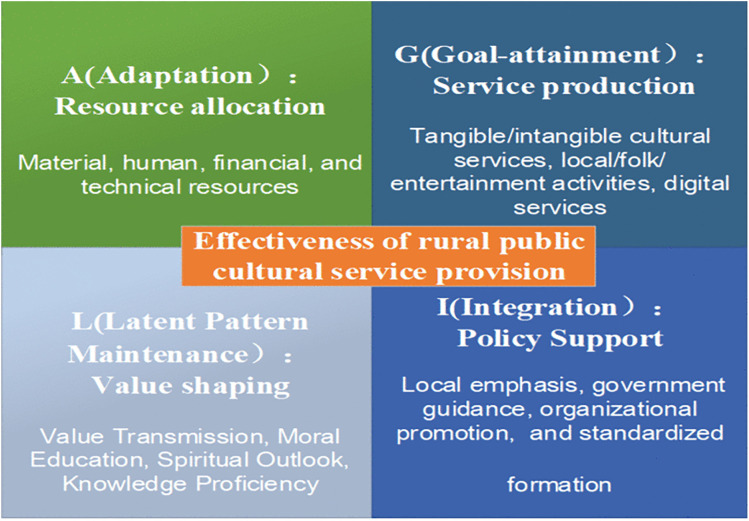
AGIL-based framework for analyzing rural public cultural service effectiveness.

Guided by the AGIL paradigm, this study operationalizes its dimensions to analyze the effectiveness of rural public cultural service provision (see Fig 1) as follows: (1) Dimension A (adaptation) refers to the resource allocation for rural public cultural service provision, which involves adjusting the input and distribution of resources such as technology, talent, material, and financial support according to needs, thereby ensuring effective provision of rural public cultural services. (2) Dimension G (goal-attainment) refers to the service production of rural public cultural services, which involves providing the public with the necessary cultural resources, cultural activities, and public cultural service products. (3) Dimension I (integration) refers to the policy support of rural public cultural service provision, which involves the government coordinating, guiding, and overseeing the construction of the public cultural service system, as well as formulating and improving cultural policies and regulations to provide intellectual support. (4) Dimension L (latent pattern maintenance) refers to value shaping, which involves the transmission of cultural values and norms through public cultural services, such as organizing community festivals that reinforce local traditions or providing educational programs that promote social cohesion. This process cultivates shared cultural values, fosters social belonging, and strengthens group consciousness among rural populations.

**Fig 2:**
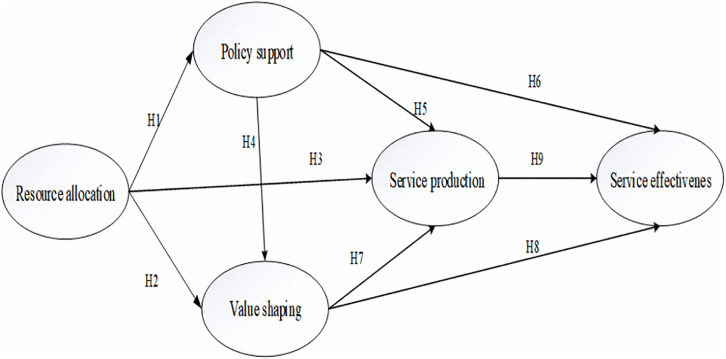
Theoretical model of factors influencing rural public cultural service effectiveness.

Building on this framework and relevant research, this study applies the AGIL model to analyze the effectiveness of rural public cultural service provision and constructs an analytical framework for the factors influencing its effectiveness. On the one hand, the framework utilizes this theory to clarify the relationship between each dimension and the effectiveness of rural public cultural service provision. Among these, “resource allocation” and “service production” are the foundational dimensions, while “value shaping” and “policy support” are the functional dimensions. Resource investment and allocation underpin the rural public cultural service provision system. The top-level design of rural public cultural services determines the value logic of public cultural services, which subsequently guides and regulates the mainstream societal values shaping residents’ cultural lives. Ultimately, through the effective output of public cultural products and services, the coupling of supply and demand in rural public cultural services is realized. Within this research analytical framework, “service effectiveness” serves as the core manifestation and ultimate measure of the system’s “goal-attainment” (G) function. It refers to the quantity of service outputs (G’s direct outputs) and comprehensively reflects the overall outcomes of services in meeting needs (realization of “adaptability” A), aligning with policy orientations (implementation of “integration” I), and permeating core values (maintenance of “latent pattern” L). Therefore, it is a composite outcome variable that integrates output performance, need fulfillment, and social value realization. On the other hand, based on this theory and related research findings, the elements of rural public cultural service provision are integrated, and the specific constituent elements of each functional dimension are preliminarily established. As a result, a theoretical model for enhancing the effectiveness of rural public cultural service provision is established (see Fig 2).

### Resource allocation and policy support, value shaping, service production

Resource investment and allocation form the foundation for effective service provision, with resources such as human, material, financial, and digital technologies providing crucial support for quality provision [21]. The integration of public cultural resources, supported by government policies, requires top-level design and coordinated management. This systematic approach is essential for establishing a comprehensive public cultural service system spanning from provincial to village levels, thereby ensuring barrier-free access [22]. In the era of digital intelligence, supported by digital technologies, leveraging digital cultural contexts and cultural carriers to fulfill social educational functions can drive the creation of new value, offering the public innovative services that enhance both their sense of accessibility and overall experience [23]. At the same time, given the current trend of public cultural needs evolving toward higher levels and greater diversity, integrating digital technologies into rural public cultural development can help consolidate existing cultural infrastructure and resources. This enables targeted implementation of digital cultural projects that align with local characteristics and needs, thereby ensuring the precise provision of public cultural products and services [24]. Establishing dedicated funds for rural public cultural services and significantly increasing fiscal investment are crucial to providing strong financial support for implementing rural public cultural activities and improving product quality and service capabilities [21]. Rural cultural leaders, artistic talents, and agricultural experts must also understand the balance between the public’s basic cultural service needs and the higher-level cultural demands of certain groups. Cultural activities, such as recreational events and agricultural technology exchanges, should be organized to meet these needs, ensuring a supply that aligns with demand [25]. Based on the above reasoning, the study proposes the following hypotheses:

H1: Resource allocation of rural public cultural services positively influences policy support.

H2: Resource allocation of rural public cultural services positively influences value shaping.

H3: Resource allocation of rural public cultural services positively influences service production.

### Policy support, value shaping, service production, and service effectiveness

Governmental top-level design, emphasis, and policy support are key driving forces. Specifically, through top-level planning, local governments incorporate citizens’ preferences and values into their action plans, guiding and leading the value orientation and spiritual essence of cultural products at the macro policy level [26]. Cultural policies encompass the balancing of cultural resources, the recognition of political values, the integration of social mechanisms, and the symbolization, aestheticization, and rationalization of various ideological advancements [27]. The authoritative values formed through these processes play a significant role in shaping rural residents’ political trust in grassroots governments [28]. From the perspective of the policy environment, sound public cultural service policies provide legal and institutional guarantees for the development of public cultural services in China [29]. Village committees should align with the policies of Rural Revitalization and cultural development, fully utilize local resources, encourage collective efforts, and organize activities such as cultural performances, sports competitions, and traditional festivals as appropriate to enrich rural public cultural spaces. From an emphasis perspective, local governments that place a high priority on public culture are more likely to break down departmental barriers, establish cross-departmental coordination mechanisms, or develop higher-level joint action frameworks, thereby improving the efficiency of public cultural services [30]. Thus, the following hypotheses are proposed:

H4: Policy support of rural public cultural services positively influences value shaping.

H5: Policy support of rural public cultural services positively influences service production.

H6: Policy support of rural public cultural services positively influences service effectiveness.

### Value shaping, service production, and service effectiveness

Integrating value symbols into rural public cultural services helps improve farmers’ spiritual and cultural life, and exerts an endogenous influence on rural values, lifestyles, action orientations, and spiritual aspirations. On the one hand, diverse and rich public cultural activities in rural areas serve as important carriers of cultural content and values. Through supply-driven demand, advanced cultural elements that reflect the values of the new era are innovatively transformed into popular public cultural services through forms, content, and other means [26]. Further, providing rural residents with external cultural products rich in modern cultural elements and civilized ideas stimulates the vitality of the rural cultural ecosystem and helps reconstruct the rural cultural system [31]. On the other hand, effective rural public cultural service provision exhibits typical capital characteristics, fostering the accumulation and appreciation of cultural capital through the utilization and continual appreciation of cultural resources. Rural public cultural services, embedded with the values and ideologies of cultural capital, facilitate the transmission between society and individuals. This process continuously shapes individual psychology and redistributes cultural resources, enhancing individuals’ understanding and recognition of their significance and value. As a result, it contributes to the construction of public faith and the enhancement of cultural identity, ultimately improving the effectiveness of rural public cultural service provision [32]. Therefore, the following hypotheses are proposed:

H7: Value shaping rural public cultural services positively influences service production.

H8: Value shaping rural public cultural services positively influences service effectiveness.

### Service production and service effectiveness

Rural public cultural services offer a rich and diverse range of provisions, including the supply of public cultural resources, activities, products, and services. Currently, precise supply is the prerequisite for effective allocation of public cultural resources, creating a positive supply-demand cycle and coupling. It mitigates issues such as inefficient or ineffective service provision and the over-complication of rural cultural governance [33,34]. In terms of cultural service provision, rural public cultural services urgently need to actively explore and effectively leverage local cultural traditions to meet the diverse cultural needs of farmers. This will facilitate precise supply-demand matching and promote the high-quality provision of rural public cultural services. Therefore, local governments should organically combine the inheritance and protection of traditional culture with the provision of cultural services. By developing and utilizing local cultural resources—such as folk culture, red culture, traditional crafts, and both tangible and intangible cultural heritage—local governments can create distinctive, diverse, and locally adapted public cultural service products and services, thereby promoting specialized, diverse rural public cultural services [35]. In addition, “interweaving” intangible cultural heritage with the public cultural service system is another effective way to improve the quality and efficiency of public cultural services [36]. At the same time, content related to ideology or intangible technologies serves a guiding role in enhancing the level of basic public cultural services and expanding the types of services offered [37]. Thus, the following hypothesis is proposed:

H9: Service production of rural public cultural services positively influences service effectiveness.

### Research design

This study was conducted by an interdisciplinary research team comprising scholars from sociology, public administration, and cultural studies. The core team consisted of two authors of this paper and three trained research assistants. All members possessed experience in rural research and were proficient in the local dialect (Sichuanese), ensuring smooth communication and cultural adaptation of survey instruments. To ensure data collection consistency and minimize interviewer bias, all members underwent specialized training prior to fieldwork. Training covered (1) research objectives and theoretical framework; (2) standardized procedures for questionnaire distribution and obtaining informed consent; and (3) techniques for conducting neutral, non-leading interviews when clarifying questions with respondents. The project leader supervised all phases of fieldwork and data entry.

Based on the AGIL model, this study constructed an indicator framework for efficiency. An initial measurement scale for the factors influencing the improvement of effectiveness was designed based on the four AGIL dimensions: adaptation (A), goal-attainment (G), integration (I), and latent pattern maintenance (L). At the same time, to ensure the rationality and validity of the scale items, a pre-survey was conducted with 200 villagers from several villages (communities) in Yibin and Ya’an cities. The data were analyzed using SPSS 23.0 for item analysis, reliability, and validity tests, and exploratory factor analysis. Unsuitable items were removed, resulting in the final scale. The scale for the factors influencing the effectiveness of rural public cultural service provision comprises four dimensions—resource allocation, service production, policy support, and value shaping—with a total of 17 observation variables. The scale for the effectiveness of rural public cultural service provision includes eight observation variables. Both scales use a five-point Likert scale for scoring. Table 1 details the measurement items corresponding to each dimension.

**Table 1:** Latent variables and observed variables of the model.

Latent variables	Observed variables	Outcome variables	Observed variables
**A: Resource allocation**(**Adaptation)**	Material resources assurance (zy1)	A: Service effectiveness (Adaptation)	Fiscal support adequacy(xn1)
	Human resources assurance (zy2)		
	Financial resources assurance(zy3)		Human resources team adaptability(xn2)
	Technical resources assurance(zy4)		
**G: Service production** **(Goal-attainment)**	Material cultural services(fw1)	G: Service effectiveness (Goal attainment)	Supply Abundance(xn3)
	Intangible cultural services(fw2)		
	Local cultural services(fw3)		
	Folk cultural activities(fw4)		Supply Satisfaction (xn4)
	Entertainment cultural activities (fw5)		
	Digital cultural services (fw6)		
**I: Policy support (Integration)**	Local emphasis (dc1)	I: Service effectiveness (Integration)	Policy and institutional implementation(xn5)
	Government guidance(dc2)		
	Organizational promotion(dc3)		Technological infrastructure development (xn6)
	Norm formation(dc4)		
**L: Value shaping (Latent Pattern Maintenance)**	Value communication(jz1)	L: Service effectiveness (Latent Pattern Maintenance)	Value Guidance Level(xn7)
	Moral education(jz2)		
	Spiritual outlook(jz3)		Cultural Environment Enhancement Level(xn8)
	Knowledge literacy(jz4)		

Given the exploratory and theoretical nature of this study and the practical difficulties in constructing a perfect sampling frame in rural Southwestern China, we employed a multistage purposive sampling strategy to obtain a diverse and information-rich sample. This non-probability sampling method is suitable for SEM research aimed at examining structural relationships between variables rather than estimating population parameters. The specific sampling process comprised three stages. First, Sichuan Province was purposefully selected as a representative province for Southwest China due to its significant socioeconomic and cultural diversity. Second, stratified purposive sampling was employed: rural areas were selected from five geographical regions—East, South, West, North, and Central Sichuan—to ensure geographic coverage. Within each region, one or two locations were selected to represent “high, “ “medium, “ and “low” levels of rural cultural development, based on expert consultation and available local statistics. Third, participants (including farmers, grassroots cadres, and community members) were recruited at the village level with the assistance of local liaisons, striving for a relatively balanced distribution across gender, educational background, and occupation. Although this approach did not yield a statistically representative sample, it covered the diverse contexts necessary for testing theoretical models. Ultimately, the study distributed 835 questionnaires across rural areas in Chengdu, Yibin, Neijiang, Suining, Guangyuan, Mianyang, Nanchong, Ya’an, and Liangshan Prefecture in Sichuan Province. A total of 815 valid questionnaires were returned, achieving a valid response rate of 97%.

Additionally, to meet the sample size requirements for SEM analysis and ensure model robustness, we screened the 815 valid questionnaires based on two criteria to determine the final model-building sample: (1) sample size adequacy and (2) data quality screening. First, according to empirical guidelines in SEM methodology, the sample size should be at least 10–20 times the number of observed variables in the model. This study’s model included 25 observed variables (see Table 1), thus requiring a theoretical minimum sample size of 250–500. To ensure model estimation stability and avoid misinterpreting weak effects as significant in large samples, we set an initial target of “no fewer than 500 respondents.” Second, to ensure data validity and rigor, we applied the following objective data-cleaning standards to select high-quality “meaningful samples” from the 815 questionnaires. For completeness, exclude questionnaires with any missing items on key latent variables (resource allocation, service production, policy support, value shaping, service effectiveness). For response consistency testing, employ reverse-scored items or pairs of items with similar content. Questionnaires exhibiting severe inconsistencies in responses to these paired items (e.g., simultaneously selecting “strongly agree” and “strongly disagree”) were excluded because such contradictions may indicate that the respondent did not read carefully. For response pattern detection, questionnaires exhibiting obvious systematic patterns (e.g., selecting the same option for all items, alternating in a wave-like pattern) were excluded because these patterns typically represent invalid responses. For screening extreme response tendencies, while not directly discarded, we examined descriptive statistics for systematic extreme scoring (e.g., selecting only the highest or lowest scores across all items) to assess potential response bias. No such systematic extremes were identified in this sample.

Following the standardized data-cleaning process described above, we obtained 610 high-quality valid samples for subsequent SEM analysis. This sample size (610) exceeds the minimum requirement (500) and meets the stringent criterion of at least 20 times the number of observed variables (25 x 20 = 500), sufficiently ensuring the stability of model parameter estimation and statistical power. Further, we compared the distribution of key demographic variables (such as gender, age, and education level) between the 205 excluded samples and the final 610 samples. No systematic differences were found, indicating that this screening process did not introduce significant sample selection bias. The final sample retains the structural diversity and representativeness of the original population.

The reliability of the developed model was assessed using Cronbach’s alpha coefficient and composite reliability (CR), and the validity was evaluated using average variance extracted (AVE). The results from the reliability and validity analysis indicate that the Cronbach’s alpha coefficients and CR values for all indicators are greater than 0.7 (see Table 2). Generally, a Cronbach’s alpha coefficient above 0.7 indicates a high level of internal consistency for the measurement model, reflecting good reliability [38]. In addition, the AVE values in Table 2 are all greater than 0.5. With the exception of the technical infrastructure completeness variable, the standardized loadings of all other variables are above 0.7. Thus, overall, the reliability and validity of the developed model are good, although further adjustments to the model fit are required in the next steps.

**Table 2:** Model reliability and validity test.

Potential variables	Observed variables	Factor loading	Cronbach’s alpha coefficient	AVE	CR
**Resource allocation**	Material resources assurance	0.742	0.852	0.589	0.851
	Human resources assurance	0.755			
	Financial resources assurance	0.821			
	Technical resources assurance	0.749			
**Service production**	Material cultural services	0.904	0.893	0.607	0.900
	Intangible cultural services	0.904			
	Local cultural services	0.844			
	Folk cultural activities	0.756			
	Entertainment cultural activities	0.606			
	Digital cultural services	0.598			
**Policy support**	Local emphasis	0.825	0.845	0.600	0.855
	Government guidance	0.710			
	Organizational promotion	0.780			
	Norm formation	0.771			
**Value shaping**	Value communication	0.701	0.824	0.551	0.831
	Moral education	0.753			
	Spiritual outlook	0.790			
	Knowledge literacy	0.723			
**Service effectiveness**	Fiscal support adequacy(xn1)	0.772	0.886	0.525	0.894
	Human resources team adaptability	0.724			
	Supply abundance	0.850			
	Supply satisfaction	0.761			
	Policy and institutional implementation	0.846			
	Technological infrastructure development	0.375			
	Value guidance level	0.556			
	Cultural environment enhancement level	0.789			

Validity refers to the accuracy of the measurement, assessing whether the results accurately reflect the measured objects. The measurement validity of the questionnaire was established through a pre-survey. Construct validity includes both convergent validity and discriminant validity. Convergent validity emphasizes that questions measuring the same variable should fall within the same dimension. In this study, the convergent validity largely meets the requirements (see Table 2). Discriminant validity refers to the ability of the scale’s latent variables to distinguish from each other. Typically, the square root of the AVE of a latent variable should be greater than its correlation with other latent variables [39]. The data in this study demonstrate good discriminant validity (see Table 3).

**Table 3:** Discriminant validity.

	Resource allocation	Service production	Policy support	Value shaping	Service effectiveness
**Resource allocation**	0.767				
**Service production**	0.58^**^	0.779			
**Policy support**	0.756^*^	0.634^**^	0.775		
**Value shaping**	0.629^**^	0.684^**^	0.583^**^	0.742	
**Service effectiveness**	0.721^*^	0.762*	0.699^**^	0.637^**^	0.725

Note: ^**^Significantly correlated at the 0.01 level (two-tailed).

To ensure the rigor of the SEM analysis, this study conducted tests for common method bias and data normality prior to analysis. Based on these results, the validity of employing the maximum likelihood estimation (ML) method was demonstrated.

First, regarding common method variance (CMV), this study employed two methods for testing. The results of the **Harman single-factor test** indicated that the unrotated first principal component explained only 38.2% of the total variance (below the 40% critical threshold). Additionally, we constructed a model incorporating a common method factor for **Common Method Variance Control** testing. Results indicated no significant improvement in model fit (ΔCFI < 0.01), with no fundamental changes in the significance or direction of core structural paths. Both tests suggest that CMV does not pose a serious threat to the conclusions of this study.

Second, we examined the normality of the data to determine whether it satisfied the prerequisites for ML estimation. The absolute values of the univariate skewness for all observed variables were less than 2, and the absolute values of the kurtosis were less than 7, meeting the empirical criteria for approximate normal distribution. Although the Mardia coefficient from the multivariate normality test indicated significant deviation (p < 0.05), the ML estimation method remains robust under conditions of moderate skewness/kurtosis. Further, the large sample size (N = 610) helps mitigate this issue.

Third, Given the aforementioned data quality and distributional characteristics, employing ML estimation for parameter estimation is appropriate. To more robustly address potential non-normality issues, we additionally utilized the **Bootstrap method (with 2,000 repeated samples)** to obtain robust standard errors and confidence intervals for parameter estimates, thereby further enhancing the reliability of statistical inference. Thus, the data foundation and estimation methods employed in this study ensure the validity and robustness of the analytical results.

### Empirical analysis

This study enhances the fit and explanatory power of the theoretical model through careful modifications. The revision process strictly adheres to the principle of prioritizing theory, adopting only theoretically justified adjustments based on the modification index (MI). For instance, residual covariance among items measuring the same dimension was added to control for common method bias, while observed variables with factor loadings below 0.5 that compromised model reliability and validity were removed. To validate the superiority of the revised model, we systematically compared it with the initial model and partially revised models. Chi-square difference tests and information criteria (AIC, BIC) revealed that the final model significantly outperformed others across all fit indices, with path coefficients aligning with theoretical expectations. This provides both statistical and theoretical support for its status as the optimal specification. Following these theoretically grounded modifications, all model fit indices reached or approached ideal standards (see revised parameter values in Table 4), indicating the revised model effectively fits the observed data. Further, the standardized factor loadings of all retained variables exceeded 0.6 (with few exceptions), and the pattern of path coefficient significance aligned with theoretical expectations, confirming the model’s measurement validity and structural validity.

**Table 4:** The effectiveness of rural public cultural service provision fit test and modification analysis results.

Fit Index	Optimal Suggested Value	Initial Reference Value	Revised Parameter Value	Revised Fit
**X** ^**2**^ **/df**	<5	6.427	4.603	Fit
**RMSEA**	<0.08	0.094	0.077	Fit
**TLI**	>0.90	0.893	0.938	Fit
**CFI**	>0.90	0.910	0.950	Fit
**IFI**	>0.90	0.911	0.950	Fit

Significance testing of the path coefficients for the full model of factors influencing the improvement of the effectiveness of rural public cultural service provision was conducted using Amos 25.0. The results are shown in Table 5.

**Table 5:** Research hypothesis testing results.

Hypothesis	Path	Standardized Path Coefficients	CR	P-value	Validation results
**H1**	Policy support← Resource allocation	0.845	17.466	***	Supported
**H2**	Value shaping← Resource allocation	−0.003	−0.029	0.977	Not supported
**H3**	Service production← Resource allocation	0.764	16.817	***	Supported
**H4**	Value shaping← Policy support	1.083	21.511	***	Supported
**H5**	Service production← Policy support	−0.177	−1.228	0.220	Not supported
**H6**	Service effectiveness←Policy support	0.635	7.452	***	Supported
**H7**	Service production← Value shaping	0.020	0.168	0.866	Not supported
**H8**	Service effectiveness←Value shaping	0.556	3.886	***	Supported
**H9**	←Service effectiveness←	0.583	5.239	***	Supported

Note: *** p  < 0.001, ** p  < 0.01, * p  < 0.05.

Table 5 shows the testing results for the research hypotheses. First, resource allocation positively impacts policy support (H1) and service production (H3). The P-values for both paths from resource allocation to policy support and from resource allocation to service production are less than 0.05, indicating that H1 and H3 are supported. However, the path from resource-driven to value-shaping has a P-value greater than 0.05, indicating it does not reach statistical significance; thus, H2 is not supported. Second, policy support positively impacts value shaping (H4) and service effectiveness (H6). The P-values for both the paths from policy support to value shaping and service effectiveness are less than 0.05, indicating that H4 and H6 are supported. However, the path from policy support to service production has a P-value greater than 0.05, indicating that H5 is not supported. Third, value shaping positively impacts service effectiveness (H8). The P-value for the path from value shaping to service effectiveness is less than 0.05, indicating that H8 is statistically significant and supported. Fourth, service production positively impacts service effectiveness (H9). The P-value for the path from service production to service effectiveness is also less than 0.05, indicating statistical significance and supporting H9.

Table 5 also indicates that, in addition to direct effects, policy support, service production, and value shaping serve as mediating factors that indirectly improve the effectiveness of rural public cultural service provision. Therefore, this paper uses policy support, service production, and value shaping as mediating variables to test the mediating effects of the corresponding paths. The Sobel Test, Aroian Test, and Goodman Test are used to analyze mediating variable effects. When the absolute value of the Z-score exceeds 1.96, it is considered significant, indicating a mediating effect and supporting the scientific validity of the related paths [40,41]. Table 6 outlines the results.

**Table 6:** Mediating effect test.

Variable relationship	Measured variables	Standardized path coefficients standard error	Sobel test’s Z-value	Aroian test’s Z-value	Goodman test’s Z-value
**Resource allocation→ Policy support →Service effectiveness**	Resource allocation→ Policy support	0.845 0.044	7.924***	7.915***	7.933***
	Policy support→Service effectiveness	0.635 0.073			
**Resource allocation→ Service production→ Service effectiveness**	Resource allocation→ Service production	0.764 0.055	12.303***	12.296***	12.310***
	Service production→ Service effectiveness	0.583 0.022			
**Resource allocation→ Policy support →Value shaping**	Resource allocation→ Policy support	0.845 0.044	14.370***	14.361***	14.378***
	Policy support→Value shaping	1.083 0.050			
**Policy support →Value shaping→ Service effectiveness**	Policy support→Value shaping	1.083 0.050	8.894***	8.886***	8.902***
	Value shaping→Service effectiveness	0.556 0.057			

Table 6 indicates that the mediating effects of policy support, service production, and value shaping have all been validated. The Z-scores for the corresponding paths are all greater than 1.96, indicating significant mediating effects along these paths. First, policy support acts as a mediator in the impact of resource allocation on service effectiveness and on value shaping. Second, service production mediates the influence of resource allocation on service effectiveness. Third, value shaping mediates the impact of policy support on service effectiveness.

**Fig 3:**
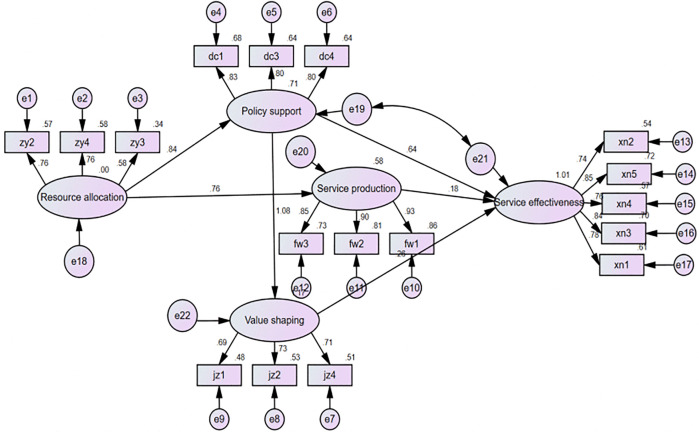
Final structural equation model for rural public cultural service effectiveness.

This study demonstrates that improving the effectiveness of rural public cultural service provision requires a multidimensional approach that integrates resources, policy, values, and service production. By applying the AGIL paradigm and SEM analysis, we have identified four key driving pathways. Based on the final structural equation model (see Fig 3, displaying standardized path coefficients), we identified four key driving paths, which are explained below with specific coefficients.

**Path 1: Targeted Resource Allocation.** This corresponds to the two pathways in Fig 3: Resource Allocation→Policy Support→Service Effectiveness and Resource Allocation→Service Provision→Service Effectiveness. In this pathway, resource allocation is the key factor, while policy support and service provision are secondary. This pathway indicates that within the current governance context of rural China, the injection of material resources serves as the primary “fuel” to initiate and lubricate the entire supply system. This finding aligns with certain characteristics of China’s “project-based” governance, in which the injection of higher-level resources often serves as the key signal triggering local government policy mobilization (I) and concrete service delivery (G). It highlights the system’s persistent, strong “resource dependency” attributes. Notably, the direct path from resource allocation to value shaping (L) is insignificant (H2 not supported), carrying profound systemic implications. It indicates that the simple injection of material resources cannot automatically or directly translate into villagers’ recognition of value and cultural internalization. As a “latent pattern maintenance” subsystem, value (L) requires more complex intermediary processes centered on meaning transmission (as reflected in Path 3 below). Thus, this insignificant pathway conversely underscores the necessity of “policy support” (I) as the “critical converter” linking resources (A) and values (L) (see Path 2). Given that service activities detached from farmers’ daily lives and lacking a local rural cultural atmosphere struggle to meet their needs, rural public cultural services should adhere to a demand-oriented principle [42]. Thus, resource allocation must be precisely tailored to the cultural needs of the rural population to ensure effective alignment between supply and demand in rural public cultural services. High-quality grassroots cultural resources and formats should be introduced and creatively transformed into cultural products and services. Concurrently, public cultural service activities that align with local characteristics and needs should be concentrated, thereby stimulating the endogenous momentum of rural public cultural services and shifting the focus of public cultural development from “delivery” to “cultivation.”

**Path 2: Comprehensive Full-factor Assurance.** This corresponds to the driving pathway in Fig 3: Resource Allocation→Policy Support→Value Shaping→Service Effectiveness. This pathway reveals the deep-seated process by which resource-driven forces, through institutional integration, are transformed into value internalization and enhanced system effectiveness. Within this framework, adaptation serves as the “fuel reservoir” initiating system survival, and the availability of its resources is a prerequisite for triggering integration functions. This relationship validates the foundational role of the “adaptation” subsystem in systems theory, which provides “necessary conditions” for other functions. Without sufficient resource inputs, the system’s integration (policy coordination) and goal attainment (service production) lack a material foundation. Integration, functioning as an “institutional converter” that links resources and values, centers on translating resources and top-level ideologies into shared values that villagers can internalize through institutional arrangements. This addresses the critical question of “how resources are transformed into recognition.” This “institutional translation” process is paramount—it answers “how resources become recognition.” Policy functions as a “meaning-conferring framework,” concretizing abstract “cultural revitalization” goals into localized value practices by organizing cultural events, defining cultural projects, and recognizing cultural exemplars. The latent pattern maintains its role as the “spiritual core” sustaining system stability and efficacy. Internalized values reduce cooperative transaction costs, stimulate participatory enthusiasm, and implicitly empower effectiveness. Refining the essence of endogenous rural cultural resources, enhancing their value, and preserving their cultural significance forge localized and distinctive cultural identities aligned with rural social development. This enables farmers to cultivate value recognition and developmental confidence in local culture during public cultural provision processes [43]. The overall significance of Path 2 underscores that “resources-institutions-values” form an inseparable, continuously interacting entity. Any attempt to bypass the “integration” (policy design) phase and directly “purchase” value recognition through resources (as indicated by H2’s non-significance) is likely to fail. Similarly, “value advocacy” lacking resource support risks becoming mere rhetoric. Thus, achieving efficient supply requires meticulously designing the complete chain of meaning production and reproduction—”resource input—institutional translation—value internalization”—ensuring positive feedback loops in the system’s “adaptation,” “integration,” and “potential pattern maintenance” functions.

**Path 3: Top-down Design Leadership.** This path corresponds to two pathways in Fig 3: Policy Support→Value Shaping→Service Effectiveness and Policy Support→Service Effectiveness. Within this pathway, policy support serves as the primary driver, while value shaping acts as a secondary factor. It vividly demonstrates how the integration (I) subsystem leads system objectives by shaping latent patterns (L) and direct interventions. The strong driving force of policy support (I) on value shaping (L) (H4 is significant) confirms that within China’s top-down cultural governance system, the transmission and integration of mainstream values heavily rely on organized policy design and promotion. Through top-level design, the government “translates” macro-level concepts like core socialist values into localized cultural policies and activities, thereby fulfilling its system-level integration function. However, a key finding is that policy support does not significantly influence service production directly (H5 not supported). While this may contradict common sense, it reveals an important buffering or decoupling mechanism within the system. It indicates that higher-level policy directives and emphasis (I) do not automatically or directly translate into diverse cultural service outputs (G) at the grassroots level. Policy implementation requires navigating complex organizational processes within the service production subsystem, potentially buffered by factors such as execution capacity, local preferences, and professional expertise. This also highlights the role of resource allocation (A) as a more direct link between policy (I) and service production (G) (H3 is significant), meaning policies often require accompanying resources to effectively drive service production. The coexistence of strong policy–value linkages and weak policy–production linkages depicts a typical phased characteristic of public cultural supply systems: “value integration takes precedence over implementation efficiency.” This indicates that the system’s integration function (unifying ideology) currently outweighs its goal attainment function (efficient output). Central to enhancing overall effectiveness may lie in strengthening institutionalized linkage channels between policy (I) and production (G), rather than relying solely on value advocacy.

**Path 4: Precise Output of Results.** This corresponds to the driving path in Fig 3: Service Supply→Service Effectiveness. This path directly aligns with the core functional output of the goal-attainment (G) subsystem within the AGIL system—namely, the direct pathway Service Production→Service Effectiveness (H9 is significant). Although concise, this path holds a pivotal position as the ultimate test and the functional loop closure within the system. It measures whether all preceding activities (A, I, L) are ultimately transformed into tangible public service outcomes. Goal-attainment (service production) serves as the system’s final outlet and efficiency indicator. The strong direct impact of service production (G) on service effectiveness demonstrates that, regardless of abundant resources, perfect policy design, or profound value advocacy, the system’s efficacy must ultimately be realized through concrete, diverse, accessible, and high-quality cultural service products and activities. This pathway validates the independent contribution of the goal-attainment subsystem: it possesses its own production logic, professional standards, and operational efficiency. Even disregarding its intermediary role for resources (A) or values (L), enhancing the quality and precision of service production itself represents the “shortest route” and “hard truth” for boosting system effectiveness. As an intermediary, service production serves as the pivotal hub connecting the system’s foundation to its ultimate objectives. Further, service production (G) plays a crucial intermediary role in how resource allocation (A) impacts effectiveness (the intermediary pathway formed by H3 and H9 is particularly significant). This underscores that the service production subsystem is not merely the final output point but also the core conversion workshop, “transforming resources into tangible benefits.” Resources (A) must undergo an effective production process (G) to realize their value. This further emphasizes that enhancing the specialization, organization, and digitization of service production is decisive for improving the entire system’s resource-to-effectiveness conversion efficiency.

## Discussion and conclusions

The existing literature has consistently noted that rural public cultural services face structural challenges, including uneven distribution [10], urban-rural disparities [11], and resource scarcity [12,13]. Moreover, cultural policies have played a pivotal role in balancing resources and symbolizing values [44]. Further to confirming the foundational driving role of “resource allocation” (A) in the Chinese context (H1, H3), this study reveals its specific transmission mechanism through SEM. By validating the potent pathway “Policy Support→Value Shaping” (H4), this research provides empirical support from China’s grassroots level for the value-guiding function of policies. Simultaneously, this study validates the significant pathway “Value Shaping →Service Effectiveness” (H8), aligning with cultural studies’ argument that shared values and cultural identity enhance community well-being and participation [45].

Overall, this study confirms that a single factor does not linearly drive the effectiveness of rural public cultural service provision, but rather stems from a structured network of synergistic operations within the AGIL four-dimensional subsystem. Specifically: (1) resource allocation (A) serves as the foundational trigger for the system, yet its efficacy must be mediated through policy integration (I) and service production (G) to be realized; (2) policy support (I) serves as the core hub, acting both as the strongest force shaping value recognition (L) (β = 1.083) and the most direct pathway to enhancing overall service effectiveness (β = 0.635). However, its direct driving effect on specific service production (G) is insignificant, revealing a disconnect in the policy-execution chain; (3) value shaping (L), while not directly driving production, makes significant indirect contributions to effectiveness by enhancing satisfaction and recognition (β = 0.556); and (4) service delivery (G), as the system’s “output end,” exerts a direct impact on effectiveness second only to policy support, with its quality and precision being critical (β = 0.583). Collectively, these findings depict the operational landscape of China’s rural public cultural service delivery system, characterized by resource dependency, policy-driven implementation, value permeation, and production fulfillment.

The contributions of this study extend beyond empirical descriptions of specific regions. The first contribution is primarily manifested in the transplantation and validation of the theoretical framework. This contribution offers insights from its theoretical framework and mechanism explanation to provide analytical tools for understanding the provision of rural public cultural services in broader contexts. The findings do not propose a universally applicable fixed pathway but rather present a set of testable variable relationships and transmission hypotheses. Future research applying this framework to other contexts should focus on examining how local institutional environments, cultural backgrounds, and developmental stages modulate the strength of each pathway within the model. Thus, the generalizability of this study primarily lies in the heuristic value of its theoretical framework and the falsifiability of its mechanism hypotheses. The second contribution is deconstructing the black box of mechanisms. Moving beyond previous research that predominantly focused on the binary factor–outcome relationship, this study employs SEM to reveal a complete chain of mediating transmission—“resources→policies→values→ production→effectiveness”—along with the uneven connection strengths between subsystems (e.g., strong “I–L” links versus weak “I–G” links). This provides a refined mechanism model for understanding the complexity of public service delivery. The third contribution is the precise measurement and positioning of the role of “value.” Empirical analysis distinguishes between the direct impact of value shaping on system efficacy and its (non-) direct influence on service production. This clarifies the functional positioning of cultural value within the supply system primarily as an efficacy enhancer and social stabilizer, rather than a production engine, thereby deepening theoretical discussions of the relationship between cultural capital and public goods provision.

The study findings have four direct policy implications for optimizing the provision of public cultural services in rural areas. First, adhere to the “dual-engine approach” to maximize effectiveness. Decision-makers should implement a dual-engine strategy that combines strong policies with substantive resources. On the one hand, continue to strengthen the guiding role of top-level design and policy integration (I). On the other hand, ensure that macro policies are backed by precise and adequate resource allocation (A) to overcome the critical bottleneck between policy emphasis and service implementation. Second, implement targeted funding to optimize resource allocation. Given the strong driving effect of resources on service production (β = 0.764), fiscal and cultural resources should shift from blanket funding toward demand-driven precision funding. Priority should be given to segments and entities (such as grassroots cultural teams and digital platforms) that directly create high-quality cultural products and services (G). Third, promote value empowerment to foster sustainable development. Value leadership (L) should be organically integrated throughout the service process, leveraging its role in enhancing effectiveness to design cultural programs with greater appeal and resonance. This requires policy (I) to transcend mere administrative directives and instead become a platform for meaning-making and resonance-building, achieving a profound shift from “delivering culture” to cultivating culture. Fourth, focus on production bottlenecks and establish a closed-loop evaluation system. Give high priority to strengthening the relatively independent subsystem of service production (G). A performance evaluation and feedback mechanism centered on service output quality, diversity, and public satisfaction should be established to form a virtuous management cycle of “resource input—production optimization—efficiency enhancement.”

### Limitations

While this study has yielded the aforementioned findings, it also has several limitations. Future research can build on this foundation to further deepen and expand the work.

Limitations of data and methods include those of causal inference in cross-sectional data. This study is based on survey data collected at a single point in time. Although the theoretical model specifies the direction of causality and the structural equation model supports the predefined pathways, cross-sectional data cannot definitively confirm the strict causal relationships and temporal order among variables. Future research may employ longitudinal tracking data or quasi-experimental designs to more robustly validate model’s dynamic causal mechanisms. Regarding the sample’s representativeness and generalizability limitations, this study employed non-probability purposive sampling, with the sample concentrated in Sichuan Province. Although the sample encompassed regions at varying levels of development, the findings—particularly the specific path coefficients—should be applied with caution when directly generalized to other regions within China or to international contexts. While the study’s findings are context-embedded, its analytical paradigm remains open to dialogue and testing. It lays the groundwork for a crucial comparative research project: examining how the AGIL system for rural public cultural service provision manifests in operational patterns that are either similar or divergent across different political-economic and cultural-geographical contexts. Future confirmatory studies should employ cross-regional, large-scale probability samples to test the model’s external validity and explore the moderating effects of regional characteristics (such as economic level and governance models) on path relationships.

There are also focused limitations of theoretical frameworks. This study rigorously follows the inherent logic of AGIL structural functionalism to construct hypotheses and models, ensuring theoretical consistency and rigorous testing. However, we must clearly recognize that any theoretical model represents a selective simplification of complex reality. While the AGIL framework’s focus enhances analytical clarity, it also implies that certain factors potentially influencing the effectiveness of rural public cultural services—such as individual villagers’ psychological and behavioral variables, or political-economic factors like power dynamics and interest distribution—have not been incorporated into this empirical model. This exclusion stems from their absence from the theory’s core concerns. The study’s primary objective was to clarify the fundamental structural relationships among the four functional modules within the system. Establishing this baseline model provides a solid comparative foundation for future research to systematically introduce the aforementioned external factors as moderating variables or competing explanations.

Limitations of measurement and conceptualization include the study’s operationalization of service effectiveness as a composite latent variable. However, the “effectiveness” of public cultural services also encompasses longer-term, more profound impacts such as social capital accumulation, enhanced community cohesion, and the sustainable transmission of cultural heritage. These long-term effects require qualitative research, longitudinal tracking, or mixed-method approaches to uncover them collectively. Although we modified the initial model according to theoretical principles, the process remains influenced by the characteristics of the sample data. The final model requires cross-validation on independent samples to confirm its stability and reproducibility, thereby enhancing the reliability of the research findings.

In summary, this study can be regarded as a robust baseline model for understanding the rural public cultural service delivery system. It clearly reveals the system’s core functional structure while explicitly delineating its boundaries. These limitations are not shortcomings of the research; rather, they precisely point to promising future research directions. These include conducting cross-regional comparisons, undertaking longitudinal studies, integrating multi-level variables, and deepening the measurement of “effectiveness.” Through continuous testing, refinement, and expansion in subsequent studies, our understanding of this complex social phenomenon will deepen and become increasingly comprehensive.

## Supporting information

S1 File This document is the questionnaire used for the empirical investigation in this study, provided as requested by the reviewers.This document is the proof of language polishing provided by a professional editing service for this article. (ZIP)
