# Supplementary material for: The effectiveness of public cultural service provision in rural areas of Southwest China: Influencing factors and driving pathways
Source: PLoS One. 2026 Feb 24;21(2):e0342794. doi: 10.1371/journal.pone.0342794 (PMC12931769; doi:10.1371/journal.pone.0342794)
Supplement: S1 File — (ZIP) [file pone.0342794.s001.zip › Supporting information/S1 File. Supporting information 1.docx]

**Survey Questionnaire on the Effectiveness of Rural Public Cultural Service Provision**

**Questionnaire ID:**

Hello! First, thank you for taking the time to complete this questionnaire! We are conducting a survey on the effectiveness of rural public cultural service provision within the context of rural revitalization. Participants for this survey are selected randomly. To ensure the authenticity and reliability of the research, the survey is anonymous, so please do not have any concerns. We kindly ask you to fill out the questionnaire carefully despite your busy schedule. Your responses are protected by the "Statistics Law," and we will keep them confidential. All content and data collected in this survey will be used solely for analytical and research purposes. There are no right, wrong, good, or bad answers; please respond based on your specific circumstances..

Thank you for your support, participation, and cooperation!

**Part I: Personal Basic Information**

**Instructions: Please select the option that best matches your actual situation for each question.**

1. Your gender is:

A. Male B. Female

2. Your education level is:

A. Primary school or below B. Junior high school

C. Senior high school D. University or above

3. Your occupation is:

A. Farming B. Migrant worker C. Self-employed D. Village cadre E. Unemployed F. Other

4. Your age is:

A. Under 18 B. 18-35 C. 35-65 D. Over 65

5. Your political affiliation is:

A. Chinese Communist Party member (including probationary member)

B. Communist Youth League member

C. Democratic Party member

D. Masses (Non-party)

**Part II: Factors Influencing Rural Public Cultural Service Provision**

Please indicate your level of agreement with the following statements based on your genuine feelings.

Rating Scale:

5 - Strongly Agree: Completely matches your feeling

4 - Agree: Fairly matches

3 - Neutral / No Strong Feeling: No particular feeling

2 - Disagree

1 - Strongly Disagree

| **Main Dimensions** | **Serial No.** | **Variable Name** | | **Question** | | **5** | | **4** | **3** | | | **2** | | | | **1** | | |  |  |  |
| --- | --- | --- | --- | --- | --- | --- | --- | --- | --- | --- | --- | --- | --- | --- | --- | --- | --- | --- | --- | --- | --- |
| **A**  **Resource Guarantee** | **Zy1** | **Material Guarantee** | | My village/community has cultural infrastructure such as rural libraries, sports and recreation activity rooms, film screening rooms, etc. | |  | |  |  | | |  | | | |  | | |  |  |  |
|  | **Zy2** | Talent Guarantee | | My village/community has dedicated staff responsible for rural cultural activities (e.g., village-level publicity and cultural officers). | |  | |  |  | | |  | | | |  | | |  |  |  |
|  | **Zy3** | Financial Guarantee | | My village/community has social funds (e.g., corporate sponsorship, personal donations) participating in rural cultural construction. | |  | |  |  | | |  | | | |  | | |  |  |  |
|  | **Zy4** | Technical Guarantee | | My village/community has a well-established public cultural online platform (e.g., WeChat official account promotions, official Douyin/TikTok promotions). | |  | |  |  | | |  | | | |  | | |  |  |  |
| **G**  **Service Production** | | **fw1** | **Material Cultural Services** | | My village/community has distinctive material cultural resources such as historical sites, ethnic villages, irrigation engineering heritage, as well as natural scenery and pastoral landscapes. | |  | |  |  | | |  | | | |  | | |  |  |
|  |  | **fw2** | Intangible Cultural Services | | My village/community has intangible cultural resources such as ethnic festivals, traditional folk customs, opera and folk arts, distinctive crafts, etc. |  | |  | |  | | | |  | | |  | | |  |  |
|  |  | **fw3** | Local Cultural Services | | The government supports my village/community in inheriting local culture (e.g., intangible cultural heritage brands, distinctive handicrafts). |  | |  | |  | | | |  | | |  | | |  |  |
|  |  | **fw4** | Folk Cultural Activities | | My village/community holds regular folk cultural activities such as dragon dances during Spring Festival, dragon boat races during Dragon Boat Festival, and year-end pig slaughtering ceremonies. |  | |  | |  | | | |  | | |  | | |  |  |
|  |  | **fw5** | Entertainment and Cultural Activities | | My village/community regularly carries out entertainment and cultural activities such as sending theatrical performances, song and dance performances, and films to the countryside. |  | |  | |  | | | |  | | |  | | |  |  |
|  |  | **fw6** | Digital Cultural Services | | My village/community encourages the online acquisition of public cultural services (e.g., promoting online reading of e-books, online arts training). |  | |  | |  | | | |  | | |  | | |  |  |
| **I**  **Policy Support** | | **dc1** | Local Emphasis | | My village/community places great importance on the construction of rural public culture. |  | |  | | |  | | | |  | | |  | | |  |
|  |  | **dc2** | Government Guidance | | The government actively guides the development of rural public culture in my village/community. |  | |  | | |  | | | |  | | |  | | |  |
|  |  | **dc3** | Organizational Promotion | | The grassroots Party organizations in my village/community frequently carry out work such as national policy publicity and public cultural education. |  | |  | | |  | | | |  | | |  | | |  |
|  |  | **dc4** | Norm Formation | | My village/community has sound management, deliberation, and other rules and regulations for public cultural services. |  | |  | | |  | | | |  | | |  | | |  |
| **L**  **Value Shaping** | | **jz1** | Value Dissemination | My village/community posts slogans, posters, and signage related to public cultural construction in public spaces. | | |  | |  | | |  | | | |  | | |  | | |
|  |  | **jz2** | Moral Education | I am satisfied with the moral education activities carried out in my village/community (e.g., selection of moral models, promotion of family virtues). | | |  | |  | | |  | | | |  | | |  | | |
|  |  | **jz3** | Spiritual Ethos | Through public cultural construction activities in recent years, the family ethos, community ethos, and rural ethos in my village/community have improved significantly. | | |  | |  | | |  | | | |  | | |  | | |
|  |  | **jz4** | Knowledge and Literacy | The overall level of vocational skills training in my village/community is good (e.g., cultivation of knowledgeable and skilled farmers). | | |  | |  | | |  | | | |  | | |  | | |

**Part III: Effectiveness of Rural Public Cultural Service Provision**

Please indicate your level of agreement with the following statements based on your genuine feelings.

Rating Scale:

5 - Strongly Agree: Completely matches your feeling

4 - Agree: Fairly matches

3 - Neutral / No Strong Feeling: No particular feeling

2 - Disagree

1 - Strongly Disagree

| **Main**  **Dimensions** | | **Serial No.** | | | **Variable Name** | | **Question** | **5** | | **4** | | **3** | **2** | **1** | |
| --- | --- | --- | --- | --- | --- | --- | --- | --- | --- | --- | --- | --- | --- | --- | --- |
| A  Service Effectiveness (Adaptation) | | **xn1** | | | Degree of Financial Support | | My village/community allocates funds to subsidize rural education (e.g., farmer technical training, compulsory education, etc.). |  | |  | |  |  |  | |
|  |  | **Xn2** | | | Adaptability of Talent Teams | | My village/community reasonably allocates staff responsible for rural cultural activities according to external environmental conditions and internal demands. |  | |  | |  |  |  | |
| G. Service Effectiveness (Goal-attainment) | | **xn3** | | | Richness of Supply | | Through public cultural construction activities in recent years, the cultural life in my village/community has become increasingly rich. | |  | |  |  |  |  |  |
|  |  | **xn4** | | | Satisfaction with Supply | | I am satisfied with the entertainment and cultural activities carried out in my village/community. | |  | |  |  |  |  |  |
| I  Service Effectiveness (Integration) | | **xn5** | Degree of Policy and System Implementation | | I believe that the rules and regulations for public culture in my village/community are implemented very effectively. | | |  | |  |  |  |  |  |  |
|  |  | **Xn6** | Degree of Technical Infrastructure Improvement | | My village/community has well-established communication infrastructure (e.g., mobile, telecom, Unicom communication base stations). | | |  | |  |  |  |  |  |  |
| L  Service Effectiveness (Latent Pattern Maintenance) | | **xn7** | Degree of Value Guidance | | I actively practice the Core Socialist Values in my daily life. | | |  | |  |  |  |  |  |  |
|  |  | **xn8** | Degree of Cultural Environment Improvement | | The social atmosphere in my village/community has greatly improved in recent years. | | |  | |  |  |  |  |  |  |

Thank you for your support, participation, and cooperation!
