# Supplementary material for: The effectiveness of public cultural service provision in rural areas of Southwest China: Influencing factors and driving pathways
Source: PLoS One. 2026 Feb 24;21(2):e0342794. doi: 10.1371/journal.pone.0342794 (PMC12931769; doi:10.1371/journal.pone.0342794)
Supplement: S1 File — (ZIP) [file pone.0342794.s001.zip › Supporting information/S2 File. Supporting information 2.pdf]

NATIVE English Editing
https:www.nativeee.com
Address: 18 East Jiuxianqiao Road,
Chaoyang District, Beijing, China
Phone: +861064125081

12th January, 2026

STATEMENT OF EDITING

This is to certify that the following document has been checked and corrected for proper English language, grammar, punctuation, spelling, and overall style by one or more of the highly-qualified, native English-speaking editors at Native English Editing.

Native English Editing provides editing and proofreading of scientific manuscripts for submission to peer-reviewed journals.

Manuscript title: The effectiveness of public cultural service provision in rural areas of Southwest China: Influencing factors and driving pathways

Date Issued: 12th January, 2026

Certificate Verification Key: 20260108100561000

Yours truly,

Graeme Brown
Co-owner Native English Editing
Email: publish@nativeee.com
Contact information of Beijing sales department in China:
Address: 18 East Jiuxianqiao Road, Chaoyang District, Beijing, China
Phone: +861064125081
Fax: +861064125081
Contact information of Australian editorial department:
Address: 42D Melrose Street, Parkdale Vic 3195, Australia
Phone: +61417560758
